# Arterial Stiffness: A Prognostic Marker in Coronary Heart Disease. Available Methods and Clinical Application

**DOI:** 10.3389/fcvm.2018.00064

**Published:** 2018-06-11

**Authors:** Vernon V. S. Bonarjee

**Affiliations:** Department of Cardiology, Stavanger University Hospital, Stavanger, Norway

**Keywords:** CAVI, PWV, arterial stiffness, prognostic marker, cardio ankle vascular index

## Abstract

Multiple biomarkers may predict short and long-term prognosis in patients with coronary heart disease, but their impact is limited when used in addition to established risk factors such blood pressure, cholesterol levels, diabetes mellitus, smoking as well as age and sex. Arteries are an integral part of the cardiovascular (CV) system. Arterial stiffness has been shown to be a predictor of cardiovascular events and mortality independent of traditional risk factors. It has also been shown that increased arterial stiffness may predict cardiovascular events in asymptomatic individuals without overt cardiovascular disease. Measuring arterial stiffness may, therefore, identify patients at risk at an early stage. Antihypertensive treatment has been shown to reduce arterial stiffness beyond its antihypertensive effect. Arterial stiffness could, therefore, be a surrogate marker of treatment that relates to prognosis. Arterial stiffness has mostly been used in research protocols, and its use as a prognostic indicator in clinical practice is still uncommon. Several methods exist that can determine parameters related to arterial stiffness, both local and in specific artery beds such as the aorta. In this brief review we present methods to evaluate arterial stiffness, their clinical utility, limitations and the advantages of a novel method, the Cardio-Ankle Vascular Index. Easier and more reproducible methods to evaluate arterial stiffness may increase the use of parameter as a risk factor for coronary heart disease in common clinical practice.

## Introduction

Factors predicting outcome in coronary heart disease may have a causal relationship to the disease, such as hypertension and hypercholesterolemia. It may also be a marker caused by the disease condition itself, including compensatory mechanisms, expressing the severity of the disease, such as elevated natriuretic peptides, and left ventricular ejection fraction. It is important to identify those at risk in order to initiate preventive treatment and closer follow-up in those at higher risk. Multiple biomarkers have been evaluated as risk markers (1) but these add limited additional information compared to traditional risk markers (2).

Arterial stiffness is a strong predictor of cardiovascular events and all-cause mortality (3–7), also in asymptomatic individuals without overt cardiovascular disease (8, 9). Risk factors related to coronary heart disease such as hypertension, diabetes mellitus, dyslipidemia, renal disease, and smoking are also associated with increased arterial stiffness (10). Arterial stiffness is an important risk factor and a useful prognostic marker for cardiovascular events, including coronary heart disease. The efficacy of blood pressure treatment and differences in efficacy between different types of antihypertensive agents could be directly evaluated by measuring arterial stiffness (11, 12). The aim of this review is to present methods to evaluate arterial stiffness, assess their usefulness as a marker of prognosis in a clinical setting, and evaluates the merits of a novel marker of arterial stiffness, the Cardio-Ankle Vascular index (CAVI).

## Structure and function of large arteries

Arteries have an outer tunica adventitia consisting of fibro-collagenous tissue and the external elastic lamina. The innermost layer is the tunica intima with fibro-collagenous tissue, internal elastic lamina and endothelium. The large arteries, which consist of the aorta, brachiocephalic trunk, carotid and iliac arteries, have a thick middle layer, the tunica media, with smooth muscle fibers and numerous layers of concentric elastic fibers. The elastic nature of these vessels allows them to distend and act as a reservoir to accommodate blood ejected by the heart in systole. Arteries distal to these such as brachial, tibial, as well as the coronary arteries are the muscular arteries, where the tunica media has predominantly a thick layer of smooth muscle fiber (13). The vasomotor properties of the smooth muscle fibers allow the arteries to contract or dilate closely regulating blood flow to their respective regions of supply.

## Propagation arterial pressure wave

During systole, the blood ejected by the left ventricle creates a wave propagating forward along the vascular wall of the large vessels distal to the heart. As the wave reaches the muscular arteries mismatched impedance results in a second smaller wave of reflected pressure traveling backwards. This wave reaches the aortic root in late systole or early diastole, augmenting aortic pressure aiding coronary flow (13–15). When the large arteries are elastic and more compliant, such as in the young, the propagation of the forward pressure wave is slower, and the augmentation occurs early in diastole. The pulse pressure (PP), which is the difference between systolic and diastolic pressure, is increased when the augmentation due to the reflected wave occurs in systole, rather than early diastole (16, 17). PP waveform is the change in pressure that occurs during a full cardiac cycle, the characteristic of which depends on where it is recorded. PP wave recorded centrally in the aorta is different for that recorded in peripheral arteries (14, 16, 17).

## Arterial stiffening

With aging, atherosclerotic changes occurs in the arteries resulting in increased stiffness. There is also an increase in wall thickness due to intimal thickening. Increased stiffness and an increase in wall thickness occur with age, also without atherosclerotic disease, due to depletion and fragmentation of elastin and the deposition of collagen in the media (18). PP changes little up to a certain age, but with increasing arterial stiffness PP increases. PP can be used as a marker of arterial stiffness, but PP measured in a peripheral artery will not be the same as that measured centrally in the aorta due to changes in PP waveform as mentioned above (16).

## Stiffness and compliance

**Figure 1:**
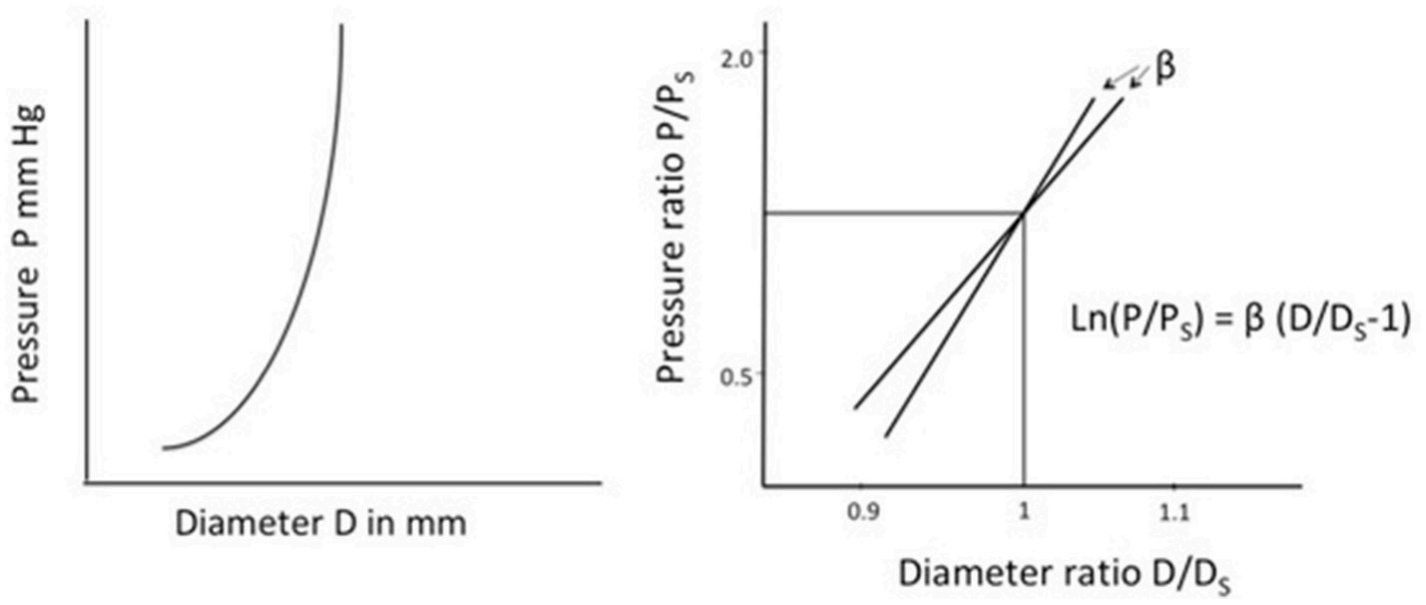
**Left**. Illustration of arterial stiffness (change in pressure/change in diameter) of an artery. Stiffness depends on the pressure at which it is measured. **Right**. Stiffness parameter β, a measure of arterial stiffness not dependent on the pressure. β is the slope and represents the stiffness of an artery. The figure illustrates β for two separate arteries. P, Measured pressure; Ps, Arbitrary standard pressure; D, Measured diameter; Ds, Diameter at standard pressure.

When pressure increases within an artery it distends the artery. The relationship between pressure and diameter is not linear. Arterial stiffness (change in pressure/change in diameter (ΔP/ΔD) gradually increases with increasing pressure (Figure 1). Whereas, vascular compliance i.e., distensibility, decreases with increasing pressure.

Arterial stiffness may be expressed as pressure-strain elastic modulus E_p_ = D(ΔP/ΔD), and compliance C_v._ = 2(ΔD/ΔP)/D (19, 20). D is diameter at pressure P, and ΔD and ΔP are changes in diameter and pressure, respectively.

The parameters E_p_ and C_v_ have different values at different pressures P and are less useful as patient specific indices on arterial stiffness and elasticity.

## Stiffness parameter β

Stiffness parameter β, suggested by Hayashi et al. (21) provides a measurement of arterial stiffness independent of blood pressure at the time of measurement within the physiological blood pressure range (63–200 mmHg). The parameter has been described in detail elsewhere (20, 22). In short, Log transformation of the pressure ratio divided by ratio of distension gives a linear curve, the angle of which represents the Stiffness parameter β (Figure 1).

This can be written as

(1) $$\beta =(D_{\text{dia}}/\Delta D)ln(P_{\text{sys}}/P_{\text{dia}})$$

β is a measure of arterial stiffness not influenced by blood pressure at the time of measurement. However, β varies between vascular beds as the properties of blood vessels vary (23). The highest being in the coronary artery, followed by carotid and renal arteries, and lowest in the abdominal and ascending aorta. A strong activation of smooth muscle cells in muscular arteries may also influence β. Stiffness parameter β has been shown to predict coronary atherosclerosis (22).

## Pulse wave velocity

The gold standard for evaluating arterial stiffness has been Pulse Wave Velocity (PWV). This is determined by recording pulse pressure waves at two different points along the arterial tree, such as between carotid and femoral, and measuring the time taken for propagation along that distance.

PWV is related to arterial stiffness. Several mathematical models and experimental studies have described this in detail (20, 24). Bramwell and Hill (25) derived the equation PWV^2^ = (ΔP/ρ) (V/ΔV) in 1926. V denotes volume, ρ the density of blood, ΔP and ΔV are changes in pressure and volume, respectively.

The volumes in the equation may be converted to area × length of a cylinder as follows:

D = vessel diameter. ΔD = Increase in diameter. L = Length

ΔV/V = {πL((D+ΔD)/2)^2^-πL(D/2)^2^}/πL(D/2)^2^

= {(D+ΔD)/2)^2^-(D/2)^2^}/(D/2)^2^

= (D^2^+ 2DΔD + ΔD^2^ – D^2^)/D^2^

= (2DΔD + ΔD^2^)/D^2^ = 2DΔD/D^2^ + ΔD^2^/D^2^

= 2ΔD/D + ΔD^2^/D^2^

As ΔD^2^/D^2^ is very small compared to 2ΔD/D it can be omitted. Thus, we may say that

ΔV/V = 2ΔD/D ie. V/ΔV = D/2ΔD

Replacing volume with diameter in the Bramwell and Hill equation we get

(2) $$\begin{array}{r} PWV^{2}=\left(\text{Δ}P/\rho \right)\left(D/2\text{Δ}D\right)i.e.,PWV^{2}\left(2\rho /\text{Δ}P\right)=D/\text{Δ}D \end{array}$$

PWV is proportional to arterial stiffness and inversely proportional to arterial compliance.

PWV has been shown to be an independent predictor of coronary heart disease and CV events (3–8, 26, 27). The predictive ability is even higher in subjects with a higher baseline CV risk (6). Automatic computerized methods to measure PWV have been validated and the methods are fairly easy to use and are reproducible (28).

PWV that include the central conduit arteries i.e., aorta and iliac arteries have demonstrated a prognostic value. PWV in the peripheral arteries such as carotid- bracial or femoro-tibial PWV have not shown such a relationship (29). However, it has been shown that Brachial-ankel (ba) PWV correlates well with aortic PWV and that most of the changes in ba-PWV following treatment is due to changes in aortic PWV (30).

The most common method used is the carotid-femoral PWV. The pressure waveform may be recorded simultaneously or sequentially and timed according to the R wave of the ECG. The length between the recording points must be measured. The transit time is usually measured as the time between the start of the upward stroke of the pulse wave at the two measuring points (31).

Although carotid-femoral-PWV is widely used there are some shortcomings. The most important is the lack of a standardized method. Some researchers use the total length between the carotid and femoral locations and divide it with the transit time to attain PWV. However, the pulse wave will move partly in opposite directions to the carotid and femoral locations. The measured distance is actually longer than the distance covered by the pulse wave during the measured transit time. A certain length such as distance from carotid to supra-sternal notch can be deducted to correct this. PWV is also affected by blood pressure changes, especially in normotensive patients (32). Standardized conditions at the time of recording are important to get reliable measurements (33). The femoral pressure waveform may be difficult to attain in obese patients, and in some subjects a reasonably correct lengths may also be difficult to measure (33). This limits its suitability in a wider clinical practice.

## Ambulatory arterial stiffness index

A more simplified surrogate indicator of arterial stiffness may be the Ambulatory Arterial Stiffness Index (AASI) derived from 24-h ambulatory blood pressure measurements (34, 35). There is a considerable diurnal variation in mean arterial pressure. In normal subjects, changes in systolic and diastolic pressure are more parallel, but in subjects with stiff, less compliant arteries, increase in mean arterial pressure results in a sharper increase in systolic than diastolic pressure. The relationship between diastolic and systolic blood pressure from all measurements in a 24-h recording can be plotted in a regression slope. AASI is determined as 1 minus the regression slope. It has been shown that AASI is an independent predictor of cardiovascular mortality, especially in normotensive patients. It seems to be a better predictor of stroke than cardiac mortalty (34, 36). Previous studies have shown some inconsistencies. AASI is highly influenced by nocturnal blood pressure drop and the relationship between AASI and other measures of arterial stiffness is weak after adjusting for confounders (37). Further studies are needed to support the usefulness of AASI as a therapeutic target in clinical practice.

## Cardio ankle vascular index

β is a measure of arterial stiffness, not dependent on the blood pressure at the time of recording, but is the property of a local segment of the artery. We may say that PWV, which measures a longer segment of the arterial tree, represents the average of the local β values from the measured segment.

Equation (2) can be written as PWV^2^ (2ρ/P_sys_-P_dia_) = D_dia_/ΔD.

Replacing D_dia_/ ΔD in equation (1) we get; β = PWV^2^ (2ρ/P_sys_-P_dia_) ln (P_sys_/P_dia_)

From this we can derive a new stiffness parameter independent of blood pressure at the time of measurement.

Heart-femoral (hf) PWV incorporates information from the entire aorta, including the base of the ascending aorta. However, non-invasive pressure recording at the aortic origin is not possible. Hasegawa introduced a method to measure transit time from the aortic origin to the femoral artery (38) by simultaneously recording the first and second heart sound on a phonocardiogram with PW recordings. Considering accessibility and quality of PW signals, Shirai et al. (39) suggested the use of heart ankle-PWV. Instead of using the common carotid artery to measure the proximal PW, he used the brachial artery.

**Figure 2:**
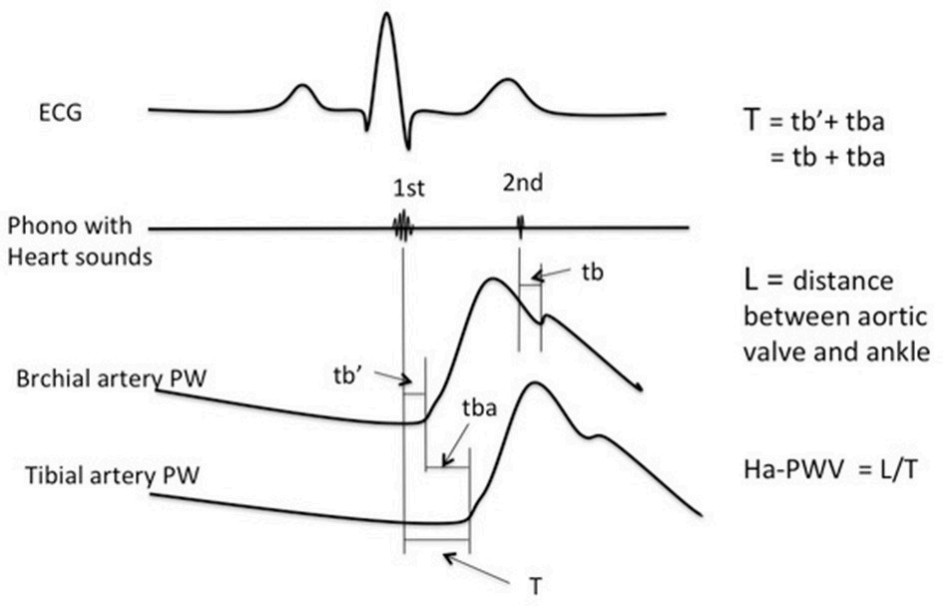
Measurement of Heart—ankle pulse wave velocity (Ha-PWV).

Here transition time (T) from the aortic origin to the ankle (tibial artery) is divided into two parts. The first is the time from the first heart sound to the start of the brachial PW (${\text{t}}_{{\text{b}}^{\prime }}$). The second is the start of brachial PW to start of ankle PW (t_ba_). T is the sum of these two (Figure 2). The second heart sound is more distinct than the first, and corresponds to the closing of the aortic valve. On the PW recording it corresponds to the dicrotic notch. Time from the second heart sound to the dicrotic notch on the brachial PW (t_b_) is practically identical to ${\text{t}}_{{\text{b}}^{\prime }}$ (39) i.e., the transit time is the same (Figure 2).

Thus, T = ${\text{t}}_{{\text{b}}^{\prime }}$ + t_ba_ = t_b_ + t_ba_. Ha-PWV is calculated as Length/T.

CAVI is defined as the stiffness parameter obtained by using ha-PWV determined as above. The actual equation is CAVI = a × (ha-PWV)^2^ (2ρ/P_sys_-P_dia_) ln (P_sys_/P_dia_)+ b.

Here a and b are two scale conversion constants to match the values of CAVI to Hasegawa's hf-PWV (39). The patent of the CAVI equation was owned by Fukuda Denshi co. Ltd (Tokyo, Japan). All calculations can be made automatically using the VeSera machines (Fukuda Denshi co. Ltd., Tokyo, Japan).

A large study with 32626 Japanese subjects, with and without CV disease, aged 20–74 years, established a baseline score for CAVI (40). The study showed that CAVI increased with age for both sexes, and that men had higher CAVI values than women of same age. Patients with CV disease had higher age and sex adjusted CAVI than those without CV disease. CAVI is easy to measure and has very good reproducibility (39, 41, 42) also in a Caucasian population (43).

Studies have compared ba-PWV with CAVI and have established that CAVI is independent of blood pressure at the time of measurement (44, 45). However, changes in contractility of smooth muscle cells in the arteries, such as by administrating alpha_1_ receptor blockers, will effect CAVI measurements (44).

In a study of 130 patients undergoing coronary angiography, CAVI correlated significantly with ba-PWV. However, only CAVI was correlated to parameters associated with atherosclerosis and only CAVI was significantly higher in patients with angina pectoris (46). In another study with 109 patients undergoing coronary angiography, CAVI was compared to carotid intima-media thickness (IMT). CAVI was significantly higher in patients with 1 vessel disease compared to no disease. It was also significantly higher in patients with 2 and 3 vessel disease compared to single vessel disease. CAVI correlated significantly to IMT but only CAVI and not IMT was independently correlated to severity of coronary artery disease (47).

There are some limitations. The reference values for CAVI are based on a Japanese population and data may indicate that the reference values may be slightly different for whites (48). CAVI is based on stiffness parameter β (21). Stiffness parameter β is calculated from diameter changes due to increase in pressure from an arbitrary standard pressure (f ex. 100 mm Hg). In CAVI this standard pressure is replaced by diastolic pressure. As diastolic pressure is not a standard pressure and varies between subjects, a certain pressure dependency is introduced. Mathematical formulas to compensate for the differences between a standard pressure and the diastolic pressure may compensate for this (49). Whether, such a correction may increase the predictive value of CAVI has not been demonstrated.

## Summary

Prognostic markers of coronary artery disease are important in order to determine who needs preventive treatment and when it needs to be initiated. Multiple biomarkers are available, but only a few of them render additional information compared to traditional risk markers. Arterial stiffness has been shown to have an independent prognostic effect on cardiovascular disease. It is also related to other markers of cardiovascular disease such as hypertension, diabetes mellitus, dyslipidemia, renal disease and smoking. Although arterial stiffness is used as a surrogate endpoint in many studies, its use is not so widespread in clinical practice. One of the main reasons is the need of standardized settings during measurements, and the dependency on blood pressure at the time of measurement. Difference in methods makes it a difficult to compare populations and obtain common reference values by which individual measurements can be matched.

Cardio Ankle Vascular Index (CAVI) is a method by which arterial stiffness can be evaluated and is independent of blood pressure at the time of measurement. It is easy to measure and the reproducibility is good. As expected, CAVI increases with age and reference values are higher in men. Studies have shown that CAVI is significantly related to coronary artery disease and parameters associated with atherosclerosis. CAVI could be used in clinical practice as a new independent risk factor for outcomes in coronary heart disease. CAVI could also be a good surrogate-endpoint to follow the effect of blood pressure treatment, and may reveal differences between drugs causing similar blood pressure reduction.

## Conclusion

Arterial stiffness is an independent prognostic indicator in coronary heart disease. CAVI, which integrates stiffness parameter β in the equation of aortic pulse wave velocity, renders a stiffness index independent of blood pressure at the time of measurement. It is easy to measure with good reproducibility. Studies indicate that CAVI is superior as a prognostic indicator compared to conventional methods of determining arterial stiffness such as bf- or ba-PWV. CAVI may, therefore, be an important and independent risk marker in patients with chronic and acute coronary heart disease.

## Author contributions

The author confirms being the sole contributor of this work and approved it for publication.

### Conflict of interest statement

The author declares that the research was conducted in the absence of any commercial or financial relationships that could be construed as a potential conflict of interest.
